# Loss of protein targeting to glycogen sensitizes human hepatocellular carcinoma cells towards glucose deprivation mediated oxidative stress and cell death

**DOI:** 10.1042/BSR20150090

**Published:** 2015-06-11

**Authors:** Rongqiang Yang, Mei Zhang, Amber Renee Gustafson, Eugenia Wang, Marsha Paulette Cole, Christine Elizabeth Schaner Tooley, Alan Cheng

**Affiliations:** * Department of Biochemistry and Molecular Genetics, University of Louisville School of Medicine, Louisville, KY 40202, U.S.A.; † Gheens Center on Aging, University of Louisville School of Medicine, Louisville, KY 40202, U.S.A.

**Keywords:** glycogen, hepatocellular carcinoma, oxidative stress, PTG

## Abstract

Protein targeting to glycogen (PTG) is a ubiquitously expressed scaffolding protein that critically regulates glycogen levels in many tissues, including the liver, muscle and brain. However, its importance in transformed cells has yet to be explored in detail. Since recent studies have demonstrated an important role for glycogen metabolism in cancer cells, we decided to assess the effect of PTG levels on the ability of human hepatocellular carcinoma (HepG2) cells to respond to metabolic stress. Although PTG expression did not significantly affect the proliferation of HepG2 cells under normal culture conditions, we determined that PTG plays an important role during glucose deprivation. Overexpression of PTG protected cells from cell death in the absence of glucose, whereas knocking down PTG further promoted cytotoxicity, as measured by the release of lactate dehydrogenase (LDH) into the media. Additionally, we demonstrated that PTG attenuates glucose deprivation induced haeme oxygenase-1 (HO-1) expression, suggesting that PTG protects against glucose deprivation-induced oxidative stress. Indeed, treating cells with the antioxidant *N*-acetyl cysteine (NAC) rescued cells from cytotoxicity caused by glucose deprivation. Finally, we showed that loss of PTG resulted in enhanced autophagy. In control cells, glucose deprivation suppressed autophagy as determined by the increase in the levels of p62, an autophagy substrate. However, in knockdown cells, this suppression was relieved. Blockade of autophagy also attenuated cytotoxicity from glucose deprivation in PTG knockdown cells. Taken together, our findings identify a novel role for PTG in protecting hepatocellular carcinoma cells from metabolic stress, in part by regulating oxidative stress and autophagy.

## Abstract (precis)

PTG is a protein that is critical for glycogen accumulation in various tissues such as the liver. Our present study shows that its loss sensitizes liver cancer cells towards metabolic and oxidative stress.

## INTRODUCTION

In mammalian cells, glycogen is the main storage form of glucose. The rate-limiting enzymes that control the synthesis and degradation of glycogen include glycogen synthase (GS) and glycogen phosphorylase (GP), respectively. Both enzymes are allosterically controlled by metabolites such as glucose-6-phosphate for GS and adenosine monophosphate for GP [1,2]. In addition, post-translational modifications such as phosphorylation, acetylation and ubiquitination can affect the activity, stability and subcellular localization of these enzymes [3,4]. For example, the phosphorylation of GS by several kinases inhibits its enzymatic activity. In contrast, GP is activated when phosphorylated on a single serine residue by phosphorylase kinase.

At the other end, the dephosphorylation of GS and GP occurs primarily through the activation of the protein phosphatase 1 (PP1), a serine/threonine protein phosphatase that is implicated in a broad range of cellular functions [5]. However, studies have shown that only a subset of PP1 molecules that are localized to glycogen particles are involved in this process. This subcellular targeting of PP1 is accomplished by glycogen targeting subunits, proteins that associate with glycogen and recruit PP1 through its interaction. In humans, at least seven glycogen-targeting subunits have been identified, encoded by the genes *PPP1R3A-G* [6]. Although they are generally implicated in glycogen metabolism, it is not entirely clear as to why so many glycogen-targeting subunits exist. Possible reasons might include differences in their tissue expression, regulation by different signals or interaction with different proteins.

We previously characterized *PPP1R3C*, encoding protein targeting to glycogen (PTG), one of the earliest known glycogen-targeting subunits. PTG is a ubiquitously expressed adaptor protein that assembles a complex involving PP1 and GS or GP [7]. By doing so, PTG stimulates glycogen synthesis, while inhibiting glycogenolysis. Over-expression of PTG in liver or fat cells markedly enhances glycogen levels [8,9]. Moreover, using gene-targeted mice, we determined the physiological importance of PTG in glycogen metabolism and in whole body glucose metabolism [10]. Loss of PTG in mice resulted in a significant decrease in glycogen stores in several tissues and altered glucose homoeostasis in mice fed a high fat diet. Deletion of the *PPP1R3A* and *PPP1R3G* genes in mice have also led to alterations in carbohydrate and lipid homoeostasis, and cumulatively suggests that glycogen-targeting subunits play crucial roles in whole body metabolism [11–13].

Although glycogen storage has long been studied as a means to control whole body glucose homoeostasis, emerging evidence indicates that its dysregulation can lead to additional disorders. Studies suggest that glycogen metabolism is important for normal brain function. For example, glycogenolysis is impaired during ischemic stroke [14]. Glycogen is stored in astrocytes, and may be critical for supporting the function of neurons [15,16]. In Lafora disease, a fatal form of epilepsy, abnormal glycogen accumulation occurs in neurons and is hypothesized to promote neuronal apoptosis. Moreover, depletion of PTG rescues multiple aspects of Lafora disease [17]. Taken together, these findings support an important biological role for glycogen metabolism in the brain beyond its function in glucose homoeostasis.

Another novel aspect of glycogen metabolism is its potential role in cancer [18,19]. Indeed, glycogen levels are up-regulated by hypoxia in several cancer cell lines. These studies also demonstrated that glycogen metabolism is enhanced in response to hypoxia. During the early period of exposure to hypoxic conditions, glycogen is accumulated. However, after prolonged exposure, glycogen is then broken down to glucose, presumably to support cell survival during low nutrient conditions. Importantly, preventing glycogen breakdown in cancer cells caused a decrease in cell proliferation. In addition, inhibition of glycogen metabolism led to cellular senescence caused by elevated oxidative stress.

Given this information, we were interested in exploring whether regulators of glycogen metabolism could be important for the survival or growth or cancer cells. We chose liver carcinoma cells since we previously studied PTG expression in rat hepatoma cells [20]. In the above study, we revealed that, PTG plays a cyto-protective role in human hepatocellular carcinoma cells in the absence of glucose. In addition, we also showed that loss of PTG increased autophagy and the induction of haeme oxygenase-1 (HO-1) in response to glucose deprivation. This new information provides insight into the cellular roles of PTG in cancer cells, and may represent a novel avenue for therapeutic intervention. Inhibiting PTG function and prohibiting glycogen metabolism in cancer cells could sensitize them to chemotherapeutic agents that produce oxidative stress and promote their death.

## MATERIALS AND METHODS

### Chemicals and antibodies

Antibodies in the present study were purchased from the following companies: V5 and green fluorescent protein (GFP) (Santa Cruz Biotechnology); aldolase A, GS, hexokinase II (HKII), Hsp70 and Hsp90, LC3A/B, pyruvate kinase M2 isoform (PKM2), 6-phosphofructo-2-kinase/fructose-2,6-biphosphatase (PFKFB)2 and PFKFB3 (Cell Signaling Technology); p62, PYGL, pyruvate dehydrogenase kinase 1 (PDHK1), HMOX-1 and P62 (Proteintech). Bafilomycin A1 (BafA1) and chloroquine (CQ) were purchased from EMD Biosciences.

### Cell culture

HepG2 and 293T cells were obtained from the American Type Culture Collection and grown in Dulbecco's modified Eagle's medium (DMEM; Cellgro) containing 10% fetal bovine serum (FBS; Cellgro). All cells were grown in the presence of antibiotics (penicillin and streptomycin) in a humidified chamber with an environment of 5% CO_2_. Cells were maintained in continuous passage by trypsinization of subconfluent cultures. For all experiments, HepG2 cells were first plated on to collagen-coated plates.

Stable cell lines were generated using a lentiviral system as previously described [21] with minor modifications. Virus was packaged and produced in 293T cells. Viral supernatants were collected after 48 h, filtered through 0.45 μM filters and concentrated using Centricon devices (Millipore). Target cells were infected with viral aliquots in the presence of polybrene overnight. Media was changed the next day and the percentage of infected cells was monitored by immunocytochemistry after an additional 24 h. Only cell populations with greater than 80% infection were used.

### Confocal fluorescence microscopy

Immunofluorescence experiments were performed as previously described [22]. In brief, cells were grown on glass coverslips coated with collagen. Following fixation with 10% formalin for 20 min at room temperature, cells were permeabilized with 0.5% Triton X-100/PBS for 5 min and then blocked with 1% bovine serum albumin and 2% goat serum for 1 h. Coverslips were incubated with primary antibodies and Alexa-Fluor conjugated secondary antibodies (Invitrogen) in blocking solution, washed and then mounted on glass slides with Vectashield (Vector Laboratories). Nuclei were stained with DAPI. Cells were imaged using a confocal fluorescence microscope (Olympus IX SLA). Images were then imported into Photoshop (Adobe Systems, Inc.) for processing.

### siRNA transfections

To knockdown PTG levels, we employed Silencer Select RNAi (Invitrogen) targeting the human gene *PPP1R3C*, and the appropriate control siRNA. For each well of a 12-well plate, 20 pmol of siRNA was transfected using 2 μl RNAiMAX (Invitrogen) in 200 μl OPTIMEM (Invitrogen) according to the manufacturer's instructions. Cells were analysed 3 or 4 days after transfection as indicated in the text.

### Preparation of cell lysates and immunoblotting

For isolation of lysates, cells were washed in ice-cold phosphate-buffered saline (PBS) and then directly lysed in SDS sample buffer. Samples were run on SDS/PAGE using Novex 4–20% gradient gels (Invitrogen). Gels were transferred to nitrocellulose, and membranes were processed for western blotting with the indicated antibodies. Membranes were imaged using the LICOR Odyssey Imaging system.

### Real-time qPCR

Total RNA was extracted from the indicated cell lines using 5Prime PerfectPure RNA isolation kits. Real-time qPCR was performed using 1-step qPCR ToughMix kit (Quanta Biosciences) on an Applied Biosystems 7900HT instrument. Glucuronidase β (GUSB) was used as the reference gene.

### Cell proliferation assay

The growth of control and gene-modified cells was determined by direct cell counting. Cells were seeded in FBS containing media at a density of 1 × 10^5^ cells/well in six-well pates. The cell numbers after culture for 24, 48, 72 and 96 h were counted by Trypan Blue exclusion assay.

### Cytotoxicity assay

Lactate dehydrogenase (LDH) cytotoxicity kit (Cayman Chemical) was utilized to determine the cell viability. PTG knockdown and overexpressed cells were seeded in a 96-well plate at a density of 5 × 10^4^ cells/well in 120 μl of culture medium. After 24 h in a CO_2_ incubator at 37°C, the cells were treated with serum free medium or glucose free medium overnight (16–18 h). Both treatments were performed in triplicate. The 96-well cell culture plate was centrifuged at 400 × *g* for 5 min. One hundred microlitres of supernatant was removed from each well and transferred into the corresponding wells of a new 96-well plate. LDH standard and reaction mixture were prepared following the manufacturer's instructions. The new 96-well plate with supernatant and standard was incubated with gentle shaking for 30 min at room temperature after adding 100 μl of reaction mixture to each well. Absorbance measurements were taken at 490 nm with a plate reader.

### Determination of glycogen

Glycogen was measured as described previously [23]. In brief, HepG2 cells were washed twice in ice-cold PBS and then snap-frozen in liquid nitrogen until further use. Cell lysis was achieved by sonication in 50 mM sodium acetate (pH 4.8). Lysates were cleared by centrifugation at 6000 *g*, and an aliquot was saved for protein determination. The assay was initiated by addition of amyloglucosidase (Sigma) and incubation for 2 h at 37°C. The amount of glycogen was determined by the amount of glucose released and measured using the Auto CII glucose kit (WAKO).

### Statistical analyses

Student's *t*test was utilized to analyse data from real-time qPCR, LDH assay and glycogen assay. Statistical significance is defined as *P*< 0.05.

## RESULTS

### Expression of PTG but not GBE1 significantly enhances glycogen storage in HepG2 cells

To determine the effect of PTG expression in HepG2 hepatocarcinoma cells, we created stable cell lines using lentivirus that expresses V5 epitope-tagged PTG. As controls, we also generated cell lines expressing GFP or the glycogen branching enzyme 1 (GBE1). Experiments were only performed on cells with over 80% infection, as determined visually by immunofluorescence staining (Figure 1A). GFP fluorescence was primarily nuclear, whereas GBE1 staining was more uniform in both the cytoplasm and nucleus. In contrast, V5 staining (for PTG) was mostly peri-nuclear.

**Figure 1:**
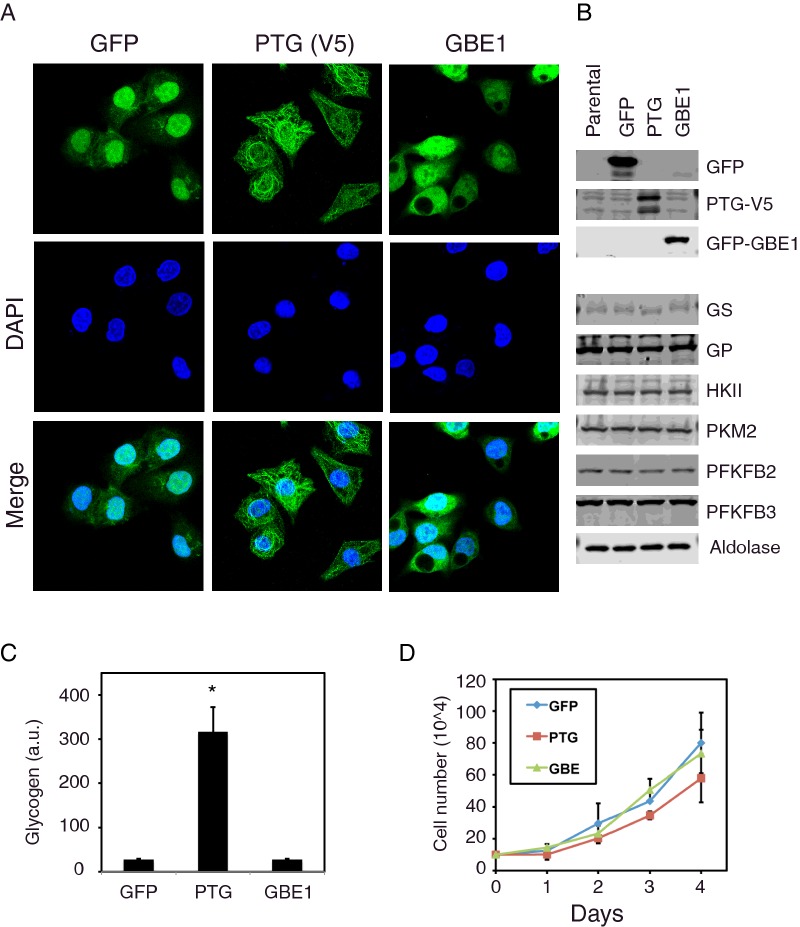
Characterization of cell lines expressing GFP, PTG or GBE1 (**A**) Immunofluorescence confocal images (40×) of stable cell lines overexpressing GFP, PTG-V5 or GFP-GBE1. Slides were stained with anti-V5 antibodies for detecting PTG. The nucleus was labelled with DAPI (blue). (**B**) Western blot showing the expression of several enzymes involved in glucose metabolism is unaltered in HepG2 cells overexpressing GFP, PTG or GBE1. (**C**) Glycogen levels are significantly increased in HepG2 stable cell lines expressing PTG, but not GFP or GBE1. Asterisk indicates *P*< 0.05 when compared with the GFP cell line. (**D**) Cell proliferation of HepG2 cells expressing GFP, PTG or GBE1 over a 4-day period.

To confirm the overexpression of PTG, we also performed western blotting (Figure 1B). Furthermore, the levels of several enzymes involved in glucose metabolism were not significantly altered by the expression of GFP, PTG or GBE1. Although overexpression of PTG did not affect the levels of the enzymes involved in glycogen metabolism (GS and GP), it did cause a 10-fold increase in glycogen levels compared with GFP or GBE1-expressing cells (Figure 1C). This increase in glycogen accumulation is similar to what has been observed in other cells or tissues [8,24].

Despite the increased accumulation of glycogen, PTG-expressing cells did not exhibit a difference in cell proliferation under normal culture conditions (Figure 1D). However, we noted that after continual passaging of cell lines, those expressing PTG began to exhibit increased proliferation compared with GFP or GBE1 expressing cells (data not shown). Therefore, for our studies, we limited the use of the cell lines within six passages.

### Expression of PTG but not GBE1 protects cells from cytotoxicity induced by glucose deprivation

Although the increased accumulation of glycogen had no effect on cell proliferation under normal culture conditions, we hypothesized that the elevated glycogen stores could provide a survival advantage under metabolically stressed conditions. To test this, we subjected the cell lines to glucose deprivation. Cells were cultured in serum-free culture media (containing 25 mM glucose) or glucose free media. After 16 h of glucose deprivation, both GFP and GBE1-expressing cells began to exhibit signs of cell death (Figure 2A). In contrast PTG-expressing cells appeared much healthier. To quantify the extent of cell death, we measured the release of LDH into the culture medium as a measure of cytotoxicity [25]. Our results demonstrate that after glucose deprivation, both GFP and GBE1-expressing cells exhibited an increase in the levels of LDH in the culture medium, when compared with cells growing in normal culture medium (Figure 2B). In contrast, PTG-expressing cells did not show a significant difference in LDH levels in the presence or absence of glucose. This suggests that over-expression of PTG can protect cells from glucose deprivation induced cytotoxicity.

**Figure 2:**
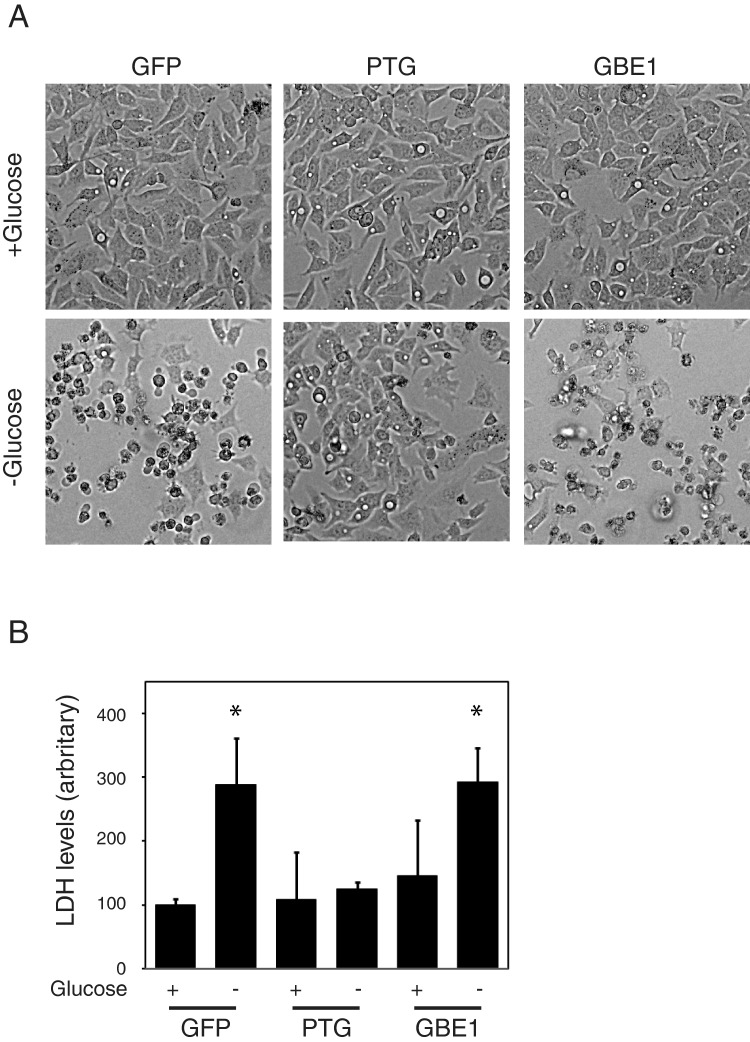
Expression of PTG prevents glucose deprivation induced cytotoxicity (**A**) Photomicrographs of HepG2 stable cell lines in the presence and absence of glucose. (**B**) LDH release into the media from HepG2 cell lines in the presence or absence of glucose. Measurements were done in triplicate and averaged. Error bars represents S.D. Asterisk indicates *P*< 0.05 when compared with the corresponding cell line in the presence of glucose.

### Expression of PTG blocks the induction of HO-1 by glucose deprivation

In cancer cells, glucose deprivation increases oxidative stress that can lead to cytotoxicity [26]. One consequence of the oxidative stress is the induction of HO-1 as a protective response [27–29]. Therefore, we assessed the level of oxidative stress by measuring the expression of *HMOX1* (the gene encoding HO-1) by quantitative real-time PCR. After glucose deprivation, both GFP and GBE1-expressing cells exhibited a significant induction of *HMOX1* gene expression (Figure 3A). In contrast, PTG-expressing cells did not. As a control, we also measured the expression of *HMOX2* (the gene encoding HO-2), and found no induction by glucose deprivation in the three cell lines (Figure 3B). Finally, western blot analysis confirmed the induction of HO-1 in GFP and GBE1-expressing cells and the lack of induction in PTG-expressing cells (Figure 3C). Together, our data suggest that over-expression of PTG can protect cells from oxidative stress induced by glucose deprivation.

**Figure 3:**
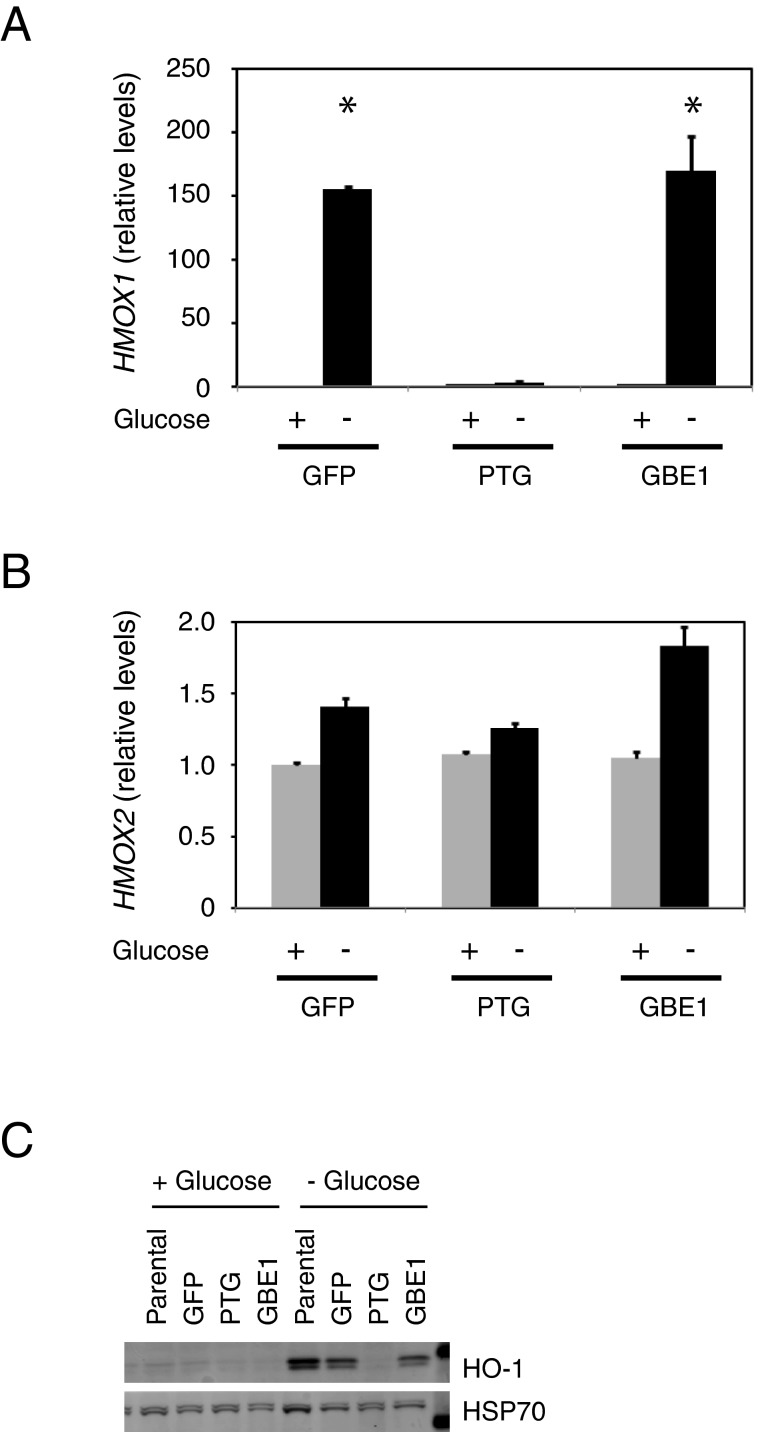
Expression of PTG prevents induction of HO-1 by glucose deprivation (**A** and **B**) Real-time qPCR analysis of *HMOX-1 and HMOX-2* in the HepG2 stable cell lines, in the presence or absence of glucose. Relative analysis was performed using *GUSB* as a reference gene. Asterisk indicates *P*< 0.05 when compared with the corresponding cell line in the presence of glucose (**C**) Western blot showing increased levels of HO-1 in GFP and GBE1 expressing cells, but not PTG expressing cells.

### Knockdown of PTG causes p21 up-regulation and enhances the effects of glucose deprivation mediated cell death

Although our data show that over-expression of PTG can protect cells from oxidative stress and cytotoxicity mediated by glucose deprivation, we wanted to know if loss of PTG would have the opposing effect. HepG2 cells express approximately half the amount of *PPP1R3C* (encoding PTG) when compared with liver tissue. To suppress PTG expression, we transfected HepG2 cells with control or *PPP1R3C* siRNA that suppresses *PPP1R3C* expression by approximately 80% by day 3 post transfection (Figure 4A). Interestingly, loss of PTG caused a morphological change that resembled senescent cells, even under normal culture conditions (Figure 4B).

**Figure 4:**
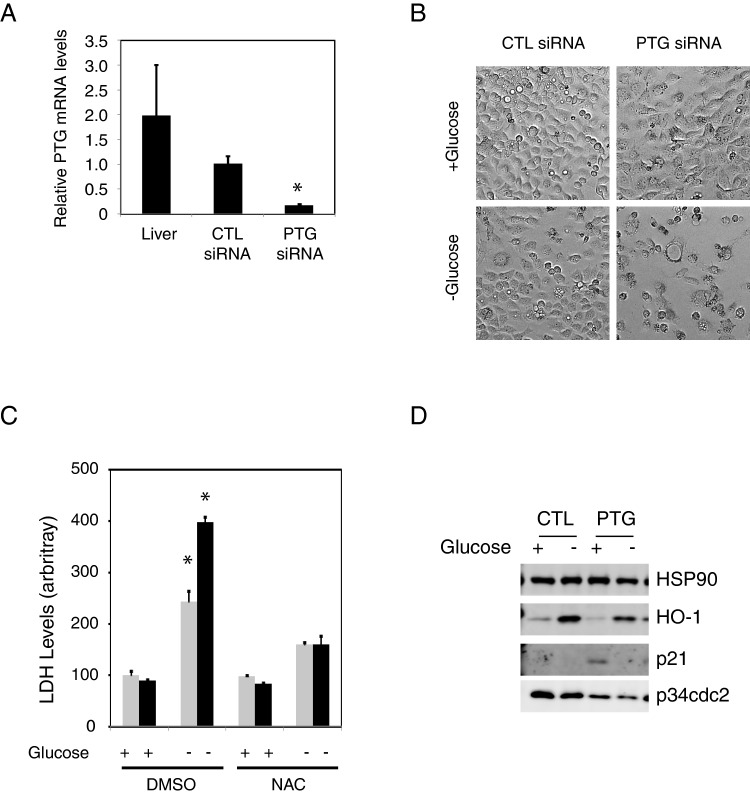
Knockdown of PTG increases cytotoxicity upon glucose deprivation (**A**) *PPP1R3C* expression in liver and HepG2 cells transfected with control and knockdown siRNA. Asterisk indicates *P*< 0.05 when compared with the control knockdown (**B**) Photomicrographs of siRNA transfected cells in the presence or absence of glucose. (**C**) LDH release into the media from transfected cells in the presence or absence of glucose, and with or without the indicated inhibitors. Measurements were done in triplicate and averaged. Error bars represents S.D. Asterisk indicates *P*< 0.05 when compared with the control knockdown cells in the presence of glucose. (**D**) Western blot analysis showing increased p21 and decreased p34cdc2 levels in the *PPP1R3C* knockdown cells.

To determine the effect of PTG knockdown during glucose deprivation, transfected cells were cultured in the presence or absence of glucose for 16 h (at 48 h post transfection). In siRNA control cells, cell death was readily apparent (Figure 4B). However, in *PPP1R3C* knockdown cells, this effect seemed more pronounced. To quantify this effect, we performed LDH measurements from the media. Our results show that loss of PTG leads to increased cytotoxicity upon glucose deprivation (Figure 4C).

To confirm that the cytotoxicity was due to oxidative stress, we also co-treated the cells with the antioxidant *N*-acetyl cysteine (NAC). In both control and *PPP1R3C* knockdown cells, treatment with NAC was able to completely block glucose deprivation induced cytotoxicity (Figure 4C). Interestingly, though, knockdown of *PPP1R3C* had no effect on HO-1 induction in the absence of glucose. This suggested that loss of *PPP1R3C* might lead to alterations in other pathways regulated by glucose withdrawal.

Because we noted that the *PPP1R3C* knockdown cells appeared senescent, we performed western blot analysis to determine the levels of the cell cycle inhibitor, p21^Cip^ (p21), a known marker of senescence [30]. In the presence of glucose, *PPP1R3C* knockdown caused a significant up-regulation of p21 (Figure 4D). However, this was not observed when cells were deprived of glucose. Additionally, *PPP1R3C* knockdown cells also exhibited decreased p34cdc2 levels, which is also consistent with a senescence phenotype [31,32].

### Knockdown of PTG enhances autophagy during glucose deprivation

Many studies have shown that nutrient deprivation signals, such as amino acid deprivation causes autophagy [33]. However, a recent study also demonstrated that glucose deprivation could inhibit autophagy in several cell lines [34]. The explanation to reconcile these observations suggested that autophagy is a high energy consuming process that is inhibited during low energy conditions such as glucose deprivation. Nevertheless, we wanted to determine if loss of PTG could affect autophagy. We transfected cells with *PPP1R3C* siRNA, and 48 h later, cultured them in the presence or absence of glucose for 16 h. Additionally, we also treated the cells with the autophagy inhibitors, CQ or BafA1 or DMSO as a control.

Our data show that treatment with either BafA1 or CQ was able to rescue PTG knockdown cells form glucose deprivation induced cytotoxicity (Figures 5A and 5B). Moreover, treatment of control knockdown cells with BafA1 had no significant effect on cytotoxicity (Figure 5B). This suggests that the increased cell death in *PPP1R3C* knockdown cells is due to increased autophagic cell death. To confirm this at the molecular level, we performed western blot analysis with the autophagic markers LC3A/B and p62 (Figure 5C). Our results show that during glucose deprivation, control knockdown cells show decreased autophagy as evidenced by increased levels of the autophagic substrate p62. In contrast, in *PPP1R3C* knockdown cells, this effect was not observed, suggesting that loss of PTG enhances autophagy during glucose deprivation.

**Figure 5:**
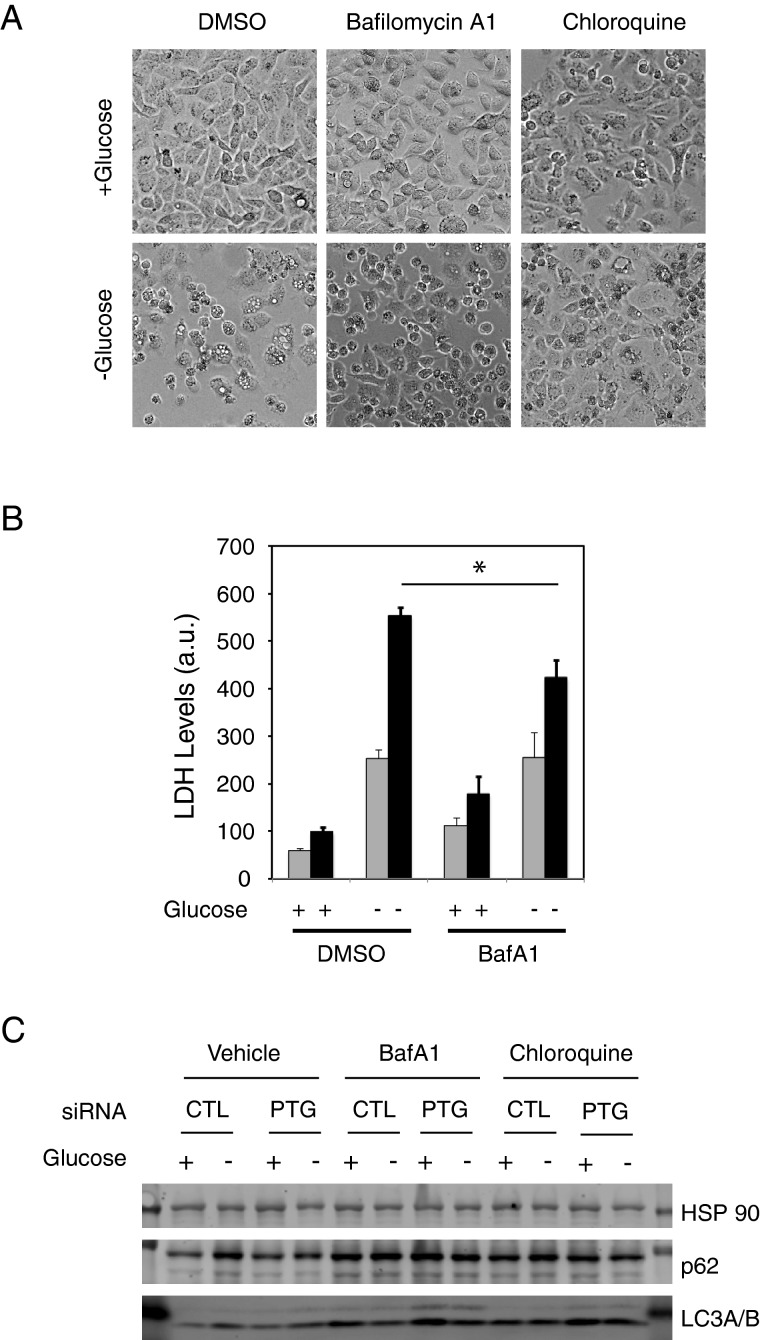
Effect of *PPP1R3C* knockdown on autophagy (**A**) Photomicrographs of *PPP1R3C* siRNA transfected cells in the presence or absence of glucose and with the indicated inhibitors. (**B**) LDH release into the media from cells in the presence or absence of glucose with or without BafA1. Light bar represents control knockdown cells, whereas dark bars represents *PPP1R3C* knockdown cells. Measurements were done in triplicate and averaged. Error bars represents S.D. Asterisk indicates *P*< 0.05 when comparing *PPP1R3C* knockdown cells in the absence of glucose with and without BafA1. (**C**) Western blot analysis of the autophagic markers LC3A/B and p62 during glucose deprivation, with or without the indicated inhibitors.

We did not observe any significant changes in LC3A/B levels upon glucose withdrawal. However, LC3A/B can experience a rapid turnover. To determine if there was a change in autophagic flux, we also performed western blots with cells treated with BafA1 and CQ (Figure 5C). Our results show that in the presence of either of these inhibitors, LC3A/B levels were increased in *PPP1R3C* knockdown cells compared with control knockdown cells. This further confirms that indeed, loss of PTG leads to in an increase in autophagy.

## DISCUSSION

Glycogen-targeting subunits are PP1 interacting proteins that play key regulatory roles in cellular glycogen levels. Furthermore, their importance on insulin sensitivity and glucose homoeostasis has been demonstrated in loss-of-function experiments in mice [10–13,35]. However, in addition to the classical insulin-responsive tissues (liver, muscle, heart and adipose) many other cell types (e.g. astrocytes, podocytes, fibroblasts) contain detectable glycogen levels. Recent evidence suggests that glycogen levels may possess important functions beyond controlling whole body metabolism. In particular, glycogen metabolism may be crucial to promote the survival of tumour cells under hypoxia [18,19,36]. Therefore, investigating the glycogen-targeting subunits of PP1 in transformed cells may uncover new insight into tumour cell metabolism.

Given the importance of PTG in the regulation of hepatic glycogen metabolism, we set out to determine its role in transformed liver cells. Our studies indicate that under conditions of glucose deprivation, PTG plays a protective role from cell death. Glucose deprivation increases oxidative stress and transformed cells are thought to be more sensitive to this [26,37]. Additionally, our studies have uncovered a novel role for PTG in autophagy. Our model suggests that PTG protects cells from cytotoxicity by attenuating oxidative stress and by preventing autophagic cell death (Figure 6). Therefore, targeting PTG may represent a new avenue to treat glycogen dependent cancers. In particular, PTG expression has been shown to be up-regulated by hypoxia in MCF-7 mammary tumour cells [38]. Therefore, our current effort is to target PTG and related pathways in breast cancer.

**Figure 6:**
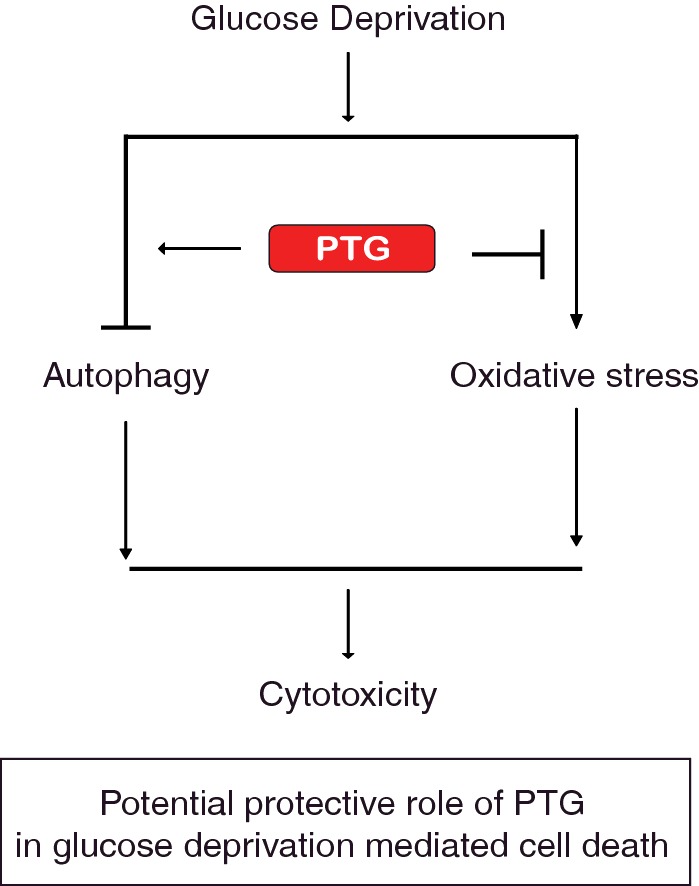
Potential protective role of PTG during glucose deprivation In the absence of glucose, PTG may play a protective role by attenuating oxidative stress and/or preventing autophagic cell death. PTG may carry out these functions through its effects on glycogen metabolism or through a yet to be identified mechanism.

Identifying the upstream regulators and downstream effectors of PTG in this aspect will be the focus of our future studies. Of interest, PTG levels are regulated by proteasomal degradation, and controlled by the dual specificity phosphatase Laforin and the E3 Ligase Malin [39–42]. Furthermore, both Laforin and Malin have been shown to play a role in autophagy and oxidative stress [43–46]. Therefore, it is possible that PTG represents an important effector downstream of Laforin/Malin in the control of these pathways.

Future efforts will also be focused on determining the mechanism by which PTG regulates autophagy. One immediate candidate to investigate would be the adenosine monophosphate-activated protein kinase (AMPK), a central regulator of metabolic pathways in both normal and transformed cells [47]. Moreover, AMPK also integrates nutrient metabolism and autophagy via the mammalian target of rapamycin complex 1 and Unc-51-like kinase 1 [48]. Interestingly, during glycogenolysis, exposure of the glycogen branches has been hypothesized to directly inhibit AMPK [49]. Therefore, PTG, via its glycogenic function may control autophagy through AMPK regulated pathways.

In summary, we have identified a protective role for PTG in human liver carcinoma cells against metabolic and oxidative stress, and a novel function in suppressing autophagy during glucose deprivation. Since both oxidative stress and autophagy play key roles in cancer cells, it will be interesting to investigate the therapeutic value of targeting PTG in various cancers by potentially sensitizing them to chemotherapeutic agents that produce oxidative stress to promote their death.

## Abbreviations

AMPK: adenosine monophosphate-activated protein kinase
BafA1: bafilomycin A1
CQ: chloroquine
GBE1: glycogen branching enzyme 1
GP: glycogen phosphorylase
GS: glycogen synthase
GUSB: glucuronidase β
HKII: hexokinase II
HO-1: haeme oxygenase-1
LDH: lactate dehydrogenase
NAC: *N*-acetyl cysteine
PDHK1: pyruvate dehydrogenase kinase 1
PFKFB: 6-phosphofructo-2-kinase/fructose-2,6-biphosphatase
PKM2: pyruvate kinase M2 isoform
PP1: protein phosphatase 1
PTG: protein targeting to glycogen
